# Laryngeal injury and dysphonia after endotracheal intubation

**DOI:** 10.25122/jml-2020-0148

**Published:** 2021

**Authors:** Ali Ahmad Al Saeg, Haitham Alnori

**Affiliations:** 1. Department of Otolaryngology, Al-Salam Teaching Hospital, Mosul, Iraq; 2. Department of Surgery, College of Medicine, University of Mosul, Mosul, Iraq

**Keywords:** laryngeal injury, dysphonia, intubation, endotracheal tube

## Abstract

We carried out a prospective case series study in order to evaluate the laryngeal complications of the endotracheal tube. Two hundred patients aged 15 years and above who were subjected to endotracheal intubation for less than 5 hours were enrolled in the study. The data were collected from the Al-Salam Teaching Hospital in Mosul, Iraq. A preoperative assessment was accomplished clinically using 70º and/or 90º Hopkins rods or fiber optic laryngoscopy. As part of the assessment, the patients' voices were recorded as well. Five to seven days after the procedure, the same assessment was repeated and compared to the preoperative data. If the postoperative examination and the voices were similar to the preoperative data, no follow-up was performed. If any abnormality was found in the larynx, the examination was repeated once weekly for one month or until the voice was recovered. In our study, five patients (2.5%) had intubation-related laryngeal injuries. The intubation period, changes in the position of the head or body of the patient during anesthesia, and the difficulty of intubation raised the possibility of laryngeal injuries. In general, intubation is a safe procedure; however, a laryngeal injury may appear as a rare complication. We found that there is a relation between the intubation period, changing the position of the patient during intubation, and difficulty of intubation with the occurrence of laryngeal injury.

## INTRODUCTION

Yearly, as part of routine anesthetic care, many patients undergo airway instrumentation and manipulation. However, with any general anesthesia, anesthesiologists need to secure the airway and provide positive pressure ventilation if the respiratory muscles become paralyzed [1]. This can be accomplished via a laryngeal mask, endotracheal tube, or tracheostomy tube. Some laryngeal complications may arise following the intubation. Such complications include, but are not limited to, vocal fold edema, subglottic stenosis, erythema, ulceration, paralysis/paresis/bowing of the vocal cords, laryngeal scar, fibrosis, granuloma formation, glottic webs, and superior-laryngeal nerve paralysis [2]. Additionally, permanent vocal cord palsy has been reported as well [3]. Even though patients usually have a rapid recovery, patients rarely experience a long-lasting or permanent injury. Different factors that may cause tube-related injury are [3, 4]:

### A. Patient-related factors

- Complications are more likely in infants, children and adult women;
- Patients with difficult airways are more susceptible to suffer an injury and hypoxic events. Difficult intubation increases the possibility of laryngeal injury. The American Society of Anesthesiologists Task Force defined difficult tracheal intubation as follows: “when proper insertion of the tracheal tube with a conventional laryngoscope requires more than three attempts or lasts more than 10 minutes”. In 2003, this definition was updated and is the procedure “requiring multiple attempts by an experienced person”;
- Preexisting laryngeal disease or abnormality.

### B. Anesthesia-related factors

- Technical skills and crisis management capabilities of the anesthesiologist;
- Hurried intubation without adequate evaluation of the airway or preparation of the patient.

### C. Equipment-related factors

- Using the stylets and bougies can be a predisposing factor to trauma.

Preventive actions that preserve laryngeal function through the identification of modifiable risk factors may reduce the incidence of endotracheal tube-related laryngeal complications. Also, after intubation, the early identification of the acute laryngeal injury may prevent the development of any chronic complications. For patients who have experienced an acute airway injury post-intubation and have been treated with early surgery require significantly fewer procedures. Also, they may avoid undergoing an invasive open surgical reconstruction compared with those who are treated after chronic fibrotic scars [4, 5].

## MATERIAL AND METHODS

Preventive actions that preserve laryngeal function through the identification of modifiable risk factors may reduce the incidence of endotracheal tube-related laryngeal complications. Also, after intubation, the early identification of the acute laryngeal injury may prevent the development of any chronic complications. For patients who have experienced an acute airway injury post-intubation and have been treated with early surgery require significantly fewer procedures. Also, they may avoid undergoing an invasive open surgical reconstruction compared with those who are treated after chronic fibrotic scars [4, 5].

This is a case series study conducted on 200 patients who were admitted to Al-Salam Teaching Hospital from August 2018 to July 2019. One hundred twenty-six patients (63%) were males, while seventy-three (37%) were females.

In the beginning, 313 individuals were considered for our study. However, the operative time was greater than 5 hours in 36 patients, the anesthetist did not use an endotracheal tube in 58 patients, and 19 patients did not attend for follow-up postoperatively. Hence, the remaining number of patients who completed the study was 200.

### Inclusion criteria

- General anesthesia with endotracheal intubation for less than five hours;
- Elective and urgent operations;
- Patients aged 14 years and above;
- Patients willing to participate in the study.

### Exclusion criteria

- General anesthesia without endotracheal intubation;
- Operative time greater than 5 hours;
- Endotracheal intubation in the emergency room and intensive care unit;
- Patients aged below 14 years;
- Thyroid, cardiac or mediastinal surgery, and different laryngeal operations that include manipulation of the larynx or tracheostomy;
- Patients with a past laryngeal surgery.

In this study, the preoperative evaluation plan included the following steps, which were accomplished either in the otolaryngology ward for elective patients or in the anesthetic side room in the theater for urgent cases. First, informed consent was taken from the patient. Then, detailed history about previous medical diseases, previous surgical operations, and smoking habits was taken from the patient. After that, the voice of the patient was recorded and saved on a mobile phone. A questionnaire was filled out, except for the part related to anesthetic and postoperative details, which were filled out later. Visualization of the larynx was performed under local anesthetic spray using an angled Hopkins rigid endoscope (70º or 90º, 4 mm diameter, 175 mm length, 10 Watt portable light source) in the majority of patients because it is easily tolerated. Fiberoptic nasopharyngoscopy (Karl Storz, 4 mm diameter, 300 mm length) was used in the examination of some patients who could not tolerate the previous method. Operative and anesthetic details, as well as the operation type, were recorded from the information sheets of the patients; additional information was obtained from the theater staff and the anesthesiologist who inserted the endotracheal tube, especially regarding difficult intubation, need for re-intubation during operation and need for changing the patient’s position during anesthesia.

Five to seven days after the operation, we repeated the laryngoscopy and voice recording and compared it with the preoperative data to diagnose any laryngeal injury or vocal cord immobility. Any abnormal finding in the laryngeal examination was considered positive, and we repeated the examination every week for one month or until the voice returned to normal.

## RESULTS

The total number of patients included in this study was 200. The age of patients ranged from 15 to 85 years, with a Mean±SD of 38.1±16.62 years. The body mass index (BMI) of the patients ranged from 15.06 to 44.98, with a Mean±SD of 25.3±4.12 years. The operative time ranged from 10 to 300 minutes, with a mean±SD of 66.4±51.62. Regarding gender distribution of the study population, 126 (63%) were males, and 74 (37%) were females.

It is noteworthy to mention that smokers represented 45.5% of the entire study population but only 4% were alcohol drinkers. The current and previous smokers were categorized into cigarette and hookah smokers (Table 1).

**Table 1:** Percentage of cigarette and hookah smokers and alcohol drinkers in the study population [n=200].

Parameters	No.	%
**Cigarette smoking:**		
Non-smoker	142	71.0
Current smoker	55	27.5
X – smoker	3	1.5
**Hookah smoking:**		
Yes	33	16.5
No	167	83.5
**Alcohol drinking:**		
Yes	8	4.0
No	192	96.0

Considering the usual scenario of endotracheal intubation, some presented difficult intubation, while extra actions, such as re-intubation or changing position, were needed for others during anesthesia (Table 2).

**Table 2:** Additional events during the operation.

Extra-event	No.	%
**Patients who needed re-intubation**	9	4.5
**Patients with difficult intubation**	10	5.0
**Patients who needed position changes**	8	4.0

**Figure 1:**
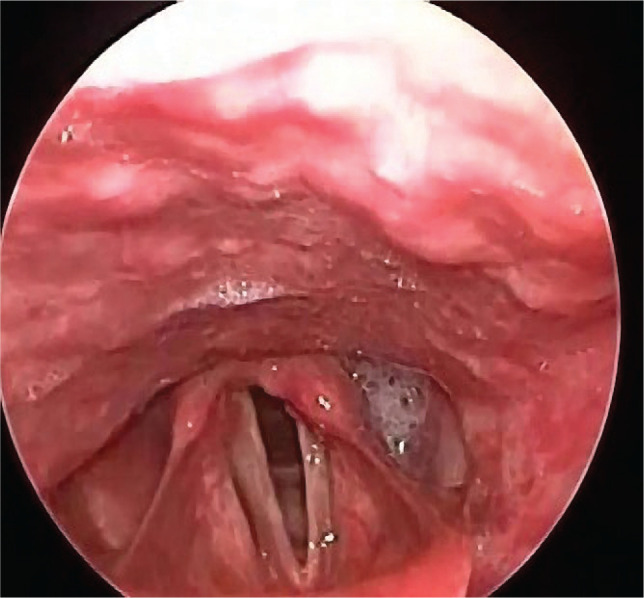
Palsy of the left vocal cord.

**Figure 2:**
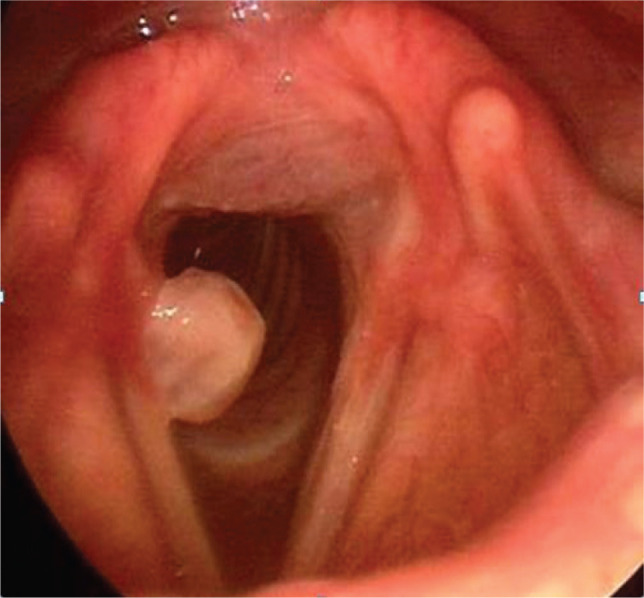
Right posterior vocal cord granuloma.

**Figure 3:**
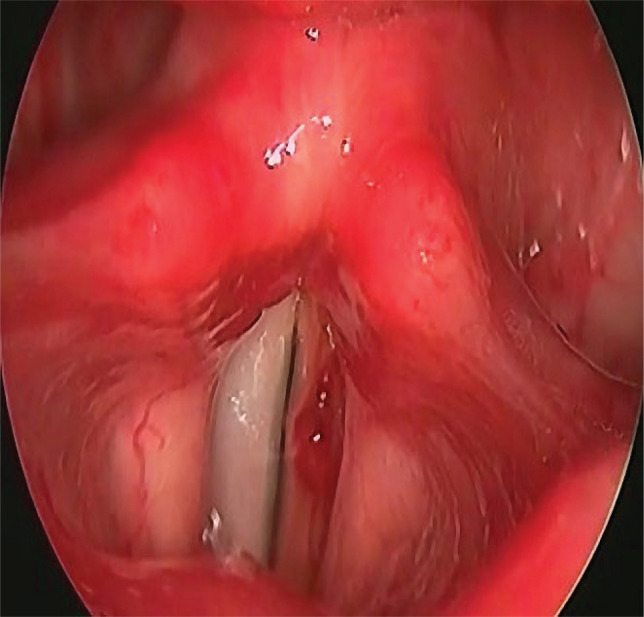
Hematoma of the left vocal cord.

Five patients presented postoperative hoarseness and/or dysphonia. The laryngeal endoscopic examination showed the following: left vocal cord palsy in one patient (Figure 1), left arytenoid dislocation in one patient, and right posterior third granuloma in one patient (Figure 2). One patient had unilateral left cord hematoma (Figure 3), which resolved after 6 weeks, and another patient had bilateral vocal cords erythema and edema, which resolved after 3 weeks.

Comparing all studied parameters between patients with positive findings to those with negative findings, we found that laryngeal injury is more common in long operations (132±45.5 minutes), which was statistically significant (p<0.05). Other socio-demographic parameters, past surgical history, smoking, drinking habits and operation class were not significant, as shown in Tables 3 and 4.

**Table 3:** Relationship between age, weight, body mass index and operative time with postoperative findings.

Parameters	Patients with positive findings [n=5] mean±SD	Patients with negative findings [n=195] mean±SD	P-value*
**Age (years)**	36.0±14.2	38.2± 16.7	0.772
**Weight (kg)**	76.6±10.1	71.2±12.8	0.352
**Body mass index (kg/m2)**	27.9±3.1	25.2±4.1	0.155
**Operative time (minutes)**	132.0±45.5	64.7±50.8	**0.004**

* Independent samples t-test was used.

**Table 4:** Relationship between postoperative findings and smoking habits.

Social habits	Positive findings		Negative findings		P-value*
	No.	%	No.	%	
**Cigarette smoking:**					
Non-smoker	3	60.0	139	71.3	
Current smoker	2	40.0	53	27.2	---**
X – smoker	0	0.0	3	1.5	
**Hookah smoking**	0	0.0	33	16.9	0.593
**Alcohol drinking**	0	0.0	8	4.1	0.999
**Total**	**5**	**100.0**	**195**	**100.0**	**---**

* Fisher’s exact test was used because of small frequency cells; ** Invalid chi-square test because of many small frequency cells.

The relation of laryngeal injury to the type of operation (elective versus urgent) is shown in Table 5. Moreover, Table 6 shows the relation between postoperative laryngeal findings and extra-events during endotracheal intubation.

**Table 5:** The relationship between laryngeal injury and the operation type.

Items	Positive findings		Negative findings		P-value*
	No.	%	No.	%	
**Operation type**					
Elective	3	60.0	150	76.9	0.335
Urgent	2	40.0	45	23.1	
**Total**	**5**	**100.0**	**195**	**100.0**	**---**

* Fisher’s exact test was used because of small frequency cells.

**Table 6:** Relationship between postoperative finding and intubation extra events during operation.

Items	Positive findings		Negative findings		P-value*
	No.	%	No.	%	
**Patients who needed re-intubation**	0	0.0	9	4.6	0.998
**Patients with difficult intubation**	3	60.0	7	3.6	**0.001**
**Patients who needed position changes**	2	40.0	6	3.1	**0.013**

* Fisher’s exact test was used because of small frequency cells.

## DISCUSSION

More than three-quarters of the patients who undergo intubation may experience hoarseness and throat discomfort in the early postoperative period. Such symptoms can be attributed to simple mucosal edema and congestion associated with intubation. These symptoms usually show a gradual improvement in the next few hours or days. After 5–7 days, the majority recover from any laryngeal complaints [5].

The age of patients from our study ranged from 15 to 85 years, with a mean age of 38.1 years. Comparison of age, weight, BMI, and intubation time between patients with positive findings to those with negative findings showed p-values of 0.772, 0.352, 0.155 and 0.004, respectively, indicating that intubation time is an important and significant factor. These results are in agreement with Brodsky *et al*., who studied patients with endotracheal intubation in intensive care unit with a mean duration of 8.2 days and found a direct relation between intubation duration and the percentage of laryngeal injuries [4].

Type and class of surgery, as well as the past surgical history, were not important parameters regarding the development of intubation events. This is logical as a laryngeal injury is expected to be related to the intubation process rather than the surgery itself [4–6]. The percentage of patients with positive findings of laryngeal injury after endotracheal intubation in our study was 2.5%. In their study from 2018, Brodsky *et al*. found that the incidence of laryngeal injuries after prolonged intubation is 83%; many of these were mild injuries, although moderate to severe injuries occurred in 13–31% of patients. This high percentage is related to the long intubation period (8.2 days) in the intensive care unit. It seems that the duration of intubation is one of the most important factors which result in laryngeal injury, even in pediatric patients [4, 7, 8].

In our study, the relationship between changing the patient’s position during operation and laryngeal injury risk was statistically significant (p=0.013), which could mean that changing the position increases the risk of laryngeal injuries. The results of the study conducted by Mota *et al*. in 2012 are in agreement with our study [9].

Re-intubation during surgery may be required for various reasons, accidental extubation being the commonest. In one study, 3 patients out of 27 who required re-intubation developed transient or permanent laryngeal injury [10]. In our study, this relation was not confirmed (p=0.998), probably due to the small sample size.

Left side laryngeal lesions and arytenoid dislocation occurred more frequently than right-sided dislocations because of the right side holding of the endotracheal tube favored by many anesthesiologists. Brodsky *et al*. found that out of 13 patients that presented with arytenoid dislocations, 8 were on the left side [4]. In our study, we identified 5 patients with laryngeal injuries, and 3 of them had left side injury, 1 had right side injury, and one had bilateral injuries.

Post-intubation injuries to the recurrent laryngeal nerve are reported more and more frequently. The incidence ranges from 0.1% to 0.2%, and it causes significant distress and morbidity in the form of hoarseness and dysphagia for the extubated patient [11]. In our series, one of the 200 patients (0.5%) had permanent vocal cord palsy. He was intubated for 5 hours for renal surgery, and the anesthesiologist described the intubation process as difficult. Intubation was attempted twice, and a stylet was not used. The reported occurrence of arytenoid cartilage dislocation is rare. It may happen even with smooth and apparently straightforward intubation [12]. The etiology is not clear, especially when the intubation process is easy.

## CONCLUSION

Generally, intubation is a safe procedure; however, a laryngeal injury may appear as an uncommon complication. We found that there is a relationship between the duration of intubation, the changing position of the patient during intubation, and the difficulty of intubation with the percentage of laryngeal injury.
